# Therapeutic time window for conivaptan treatment against stroke-evoked brain edema and blood-brain barrier disruption in mice

**DOI:** 10.1371/journal.pone.0183985

**Published:** 2017-08-30

**Authors:** Emil Zeynalov, Susan M. Jones, J. Paul Elliott

**Affiliations:** 1 Swedish Medical Center, Neurotrauma Research, Englewood, Colorado, United States of America; 2 Colorado Brain and Spine Institute, Englewood, Colorado, United States of America; University of South Florida, UNITED STATES

## Abstract

**Background:**

Ischemic stroke is often complicated by brain edema, disruption of blood-brain barrier (BBB), and uncontrolled release of arginine-vasopressin (AVP). Conivaptan, a V1a and V2 receptor antagonist, reduces brain edema and minimizes damage to the blood-brain barrier after stroke. Most stroke patients do not receive treatment immediately after the onset of brain ischemia. Delays in therapy initiation may worsen stroke outcomes. Therefore, we designed a translational study to explore the therapeutic time window for conivaptan administration.

**Methods:**

Mice were treated with conivaptan beginning 3, 5, or 20 hours after 60-minute focal middle cerebral artery occlusion. Treatments were administered by continuous IV infusion for a total of 48 hours. Brain edema and blood-brain barrier (BBB) disruption were evaluated at endpoint.

**Results:**

Conivaptan therapy initiated at 3 hours following ischemia reduced edema in the ipsilateral hemisphere, which corresponded with improvements in neurological deficits. Stroke-triggered BBB disruption was also reduced in mice when conivaptan treatments were initiated at 3 hours of reperfusion. However, 5 and 20-hour delays of conivaptan administration failed to reduce edema or protect BBB.

**Conclusion:**

Timing of conivaptan administration is important for successful reduction of brain edema and BBB disruption. Our experimental data open new possibilities to repurpose conivaptan, and make an important “bench-to-bedside translation” of the results into clinical practice.

## Introduction

Stroke presents enormous challenges for medical professionals, patients and their families. Stroke triggers a wide range of pathophysiological complications due to destruction of cells in the ischemic core, as well as brain edema and blood-brain barrier (BBB) disruption [1]. Brain edema develops soon after the onset of ischemic insult and causes elevation of intracranial pressure (ICP) [1], greatly worsening chances for recovery. BBB breakdown results in extravasation of blood contents into the extracellular space of the brain [2]. Both brain edema and BBB disruption contribute to additional damage of brain tissue and need to be addressed before the injury is further aggravated [3]. It has been shown that arginine-vasopressin (AVP) released after ischemic brain injury may exacerbate brain edema [4]. AVP-induced activation of V1a receptors triggers vasoconstriction [5] and platelet activation [6], and stimulation of V2 receptors results in water retention in the body [7]. We have previously shown that the AVP receptor blocker conivaptan, which acts on V1a and V2 receptors, reduces brain edema formation and BBB breakdown in mice when administered immediately at reperfusion [8]. This suggests a promising treatment strategy against brain edema and BBB disruption in stroke patients [9].

In clinical settings, stroke patients begin to receive their treatment only after confirmation of the diagnosis, which may take several hours after the onset of ischemic brain injury. Therefore, evaluation of the therapeutic time window for administration of conivaptan against brain edema and BBB disruption would present great translational value for stroke patients. Hence, we designed a study to assess the impact of delayed initiation of conivaptan treatment on formation of stroke-triggered brain edema and BBB breakdown in mice.

## Materials and methods

Experiments were carried out in accordance with the guidelines of the NIH for the care and use of animals and were approved by the Swedish Medical Center Animal Care and Use Committee.

**Fig 1:**
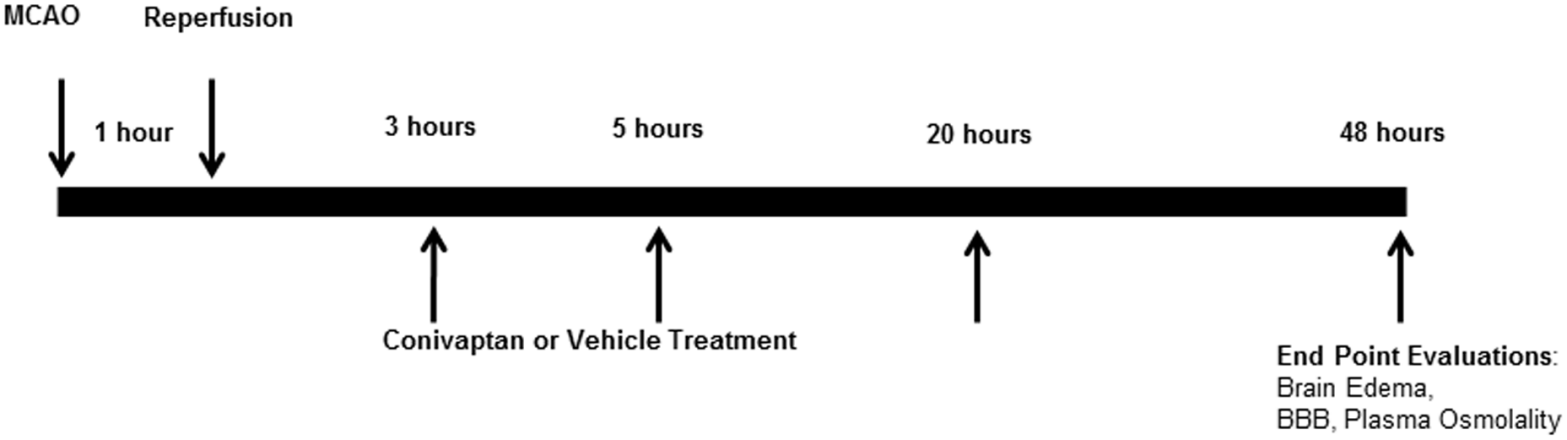
Diagram of experimental design. Mice underwent 60-minute transient focal brain ischemia by middle cerebral artery occlusion (MCAO) with reperfusion. Treatments with normal saline (NS) or conivaptan were initiated at 3, 5, or 20 hours following MCAO. At the end point (48 hours following MCAO) brain edema, BBB integrity, plasma osmolality, and neurological deficits scoring were assessed in all experimental animals.

Wild type C57BL/6 male mice (Harlan Laboratories, Inc., Indianapolis, IN), 3 months old, 26–30 g, underwent 60 minutes of transient focal brain ischemia by middle cerebral artery occlusion (MCAO) followed by reperfusion [10]. Mice were randomly assigned to treatment groups. Neurological deficit score (NDS) was assessed prior to reperfusion and at the endpoint [8, 10]. Three mice exhibited neurological deficit scores less than 2 and were excluded due to insufficient occlusion before initiation of the treatment [8]. In the first set, 63 mice were used to examine edema. In the second set, 63 mice were used to assess stroke-induced BBB breakdown. Continuous IV treatment of conivaptan 0.2 mg/mouse/day (Astellas Pharma US Inc, Deerfield, IL) or normal saline was initiated at 3, 5, or 20 hours and sustained until the 48-hour time point after MCAO. We chose the 48 hour time for the end-point of the experiment because these experiments were an extension of our earlier study in male mice [8, 10]. Females were not included. Fig 1 shows a schematic of the procedural timeline.

Each set of experiments was conducted over 3–4 months. A random generator table (www.randomizer.org) was used to determine into which treatment group each mouse was allocated on the day of surgery. Mice that died due to surgery were replaced so that final numbers were equal in each group. The n required for each endpoint (n of 10/group for edema and 5-8/group for blood brain barrier integrity) was sufficient to provide at least 0.80 power in power analysis using calculated effect sizes from our earlier studies (G*Power 3.1.7; http://www.psycho.uni-duesseldorf.de/abteilungen/aap/gpower3/).

All surgical procedures: MCA occlusion, reperfusion, and IV catheter installation, were performed under isoflurane anesthesia [8]. The middle cerebral artery (MCA) was occluded by the filament technique. Then, blood flow to the brain was restored after 1 hour of occlusion. Animals were awakened and neurological deficit scoring (NDS) was evaluated as previously described [10]. During the surgery normal body temperature was maintained within physiological range by heating pad and monitored by a thermometer attached to the rectal temperature probe. The IV line (Silastic Tubing, SIL-3-25, Strategic Applications, Inc., Lake Villa, IL) was installed into the jugular vein immediately before initiation of treatment—at 3, 5, or 20 hours of occlusion. The IV catheter was connected to an infusion pump through swivels (375/22PS, Instech Laboratories, Inc. Plymouth Meeting, PA) to ensure free movement of mice in their cages. Animals were treated with conivaptan or vehicle until the 48-hour time point after MCA occlusion. The dose and the infusion rate of 1.5 ml/kg/hour were calculated and justified based on conivaptan human doses approved by the FDA, adjusted for weight. It was not possible for the surgeon to be blinded with regard to treatment. However, endpoint indices on harvested tissue were performed without knowledge of treatment group, which was not included in labels of the harvested tissue.

Brain edema was assessed by comparing brain water content (BWC) between groups [8]. To measure brain edema the brains were removed and separated into the ipsilateral and contralateral hemispheres. Hemispheres were weighed before and after they were dried in an oven for 3 days at 100°C. Brain water content (BWC) was calculated as % H_2_O = (1-dry weight/wet weight) × 100 [8].

For BBB breakdown evaluation Evans Blue (2% in 0.9% saline; 3 ml/kg) was injected intravenously. After 2 hours brains were perfused with normal saline, removed, and dissected into left (ischemic) and right (non-ischemic) hemispheres. Each hemisphere was homogenized in 2 ml of 50% trichloroacetic acid solution. After centrifugation at 3,000 x g, the supernatants were diluted with ethanol (1:3). EB concentration was determined with a spectrophotometer at 620 nm for absorbance against a standard curve. BBB disruption data is presented as EB extravasation index which is the ratio of EB concentration in the ischemic hemisphere to that in the non-ischemic hemisphere (I/C).

To confirm the aquaretic effect of conivaptan we measured plasma osmolality in all experimental animals before mice were euthanized. Blood was aspirated directly from the heart, and osmolality (mOsmol/L) of plasma was evaluated using vapor pressure osmometry (VAPRO 5520; WESCOR, Inc, Logan UT) as described [8].

## Statistical analysis

Values for BWC and EB extravasation index are expressed as mean ± SEM. BWC was analyzed by two-way ANOVA (treatment and hemisphere as factors) with post hoc Bonferroni test. EB extravasation index was analyzed by one-way ANOVA with Bonferroni post-hoc test. Physiological indices, as well as plasma were analyzed by unpaired t-test. NDS is presented as median (with 25% and 75% quartiles) and the improvement (difference between 0 and 48 hour NDS) was analyzed by the non-parametric Kruskal-Wallis with Dunns post-hoc test. Effects were considered statistically significant at p<0.05.

## Results

**Fig 2:**
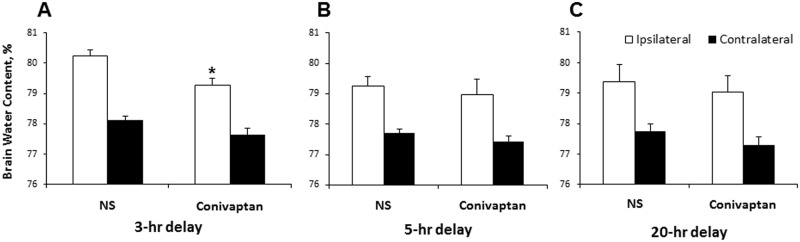
Conivaptan reduces brain edema when administered with 3 hour delay. Mice were subjected to 1-hour MCAO with reperfusion followed by IV treatment initiated at 3, 5, or 20 hours after onset of ischemia. Conivaptan treatment initiated at 3-hour delay reduced brain water content (BWC) in the ipsilateral hemisphere compared to normal saline (NS) treated mice (A). However, 5 or 20-hour delay in treatment initiation failed to reduce brain edema (B and C). Values are mean ± SEM *p ˂ 0.05 vs. NS-treated mice, n = 10 per group.

Mice treated with normal saline following MCAO showed an increase in BWC in the ipsilateral hemisphere compared to the contralateral hemisphere, Fig 2A, 2B and 2C. In contrast, conivaptan treatment beginning after 3-hour delay significantly reduced BWC values in the ipsilateral hemisphere (p < 0.05), Fig 1A. Longer delays for conivaptan treatment initiation did not reduce ipsilateral brain edema, Fig 1B and 1C. Conivaptan treatment did not affect BWC in the contralateral hemisphere, Fig 1A, 1B and 1C.

**Fig 3:**
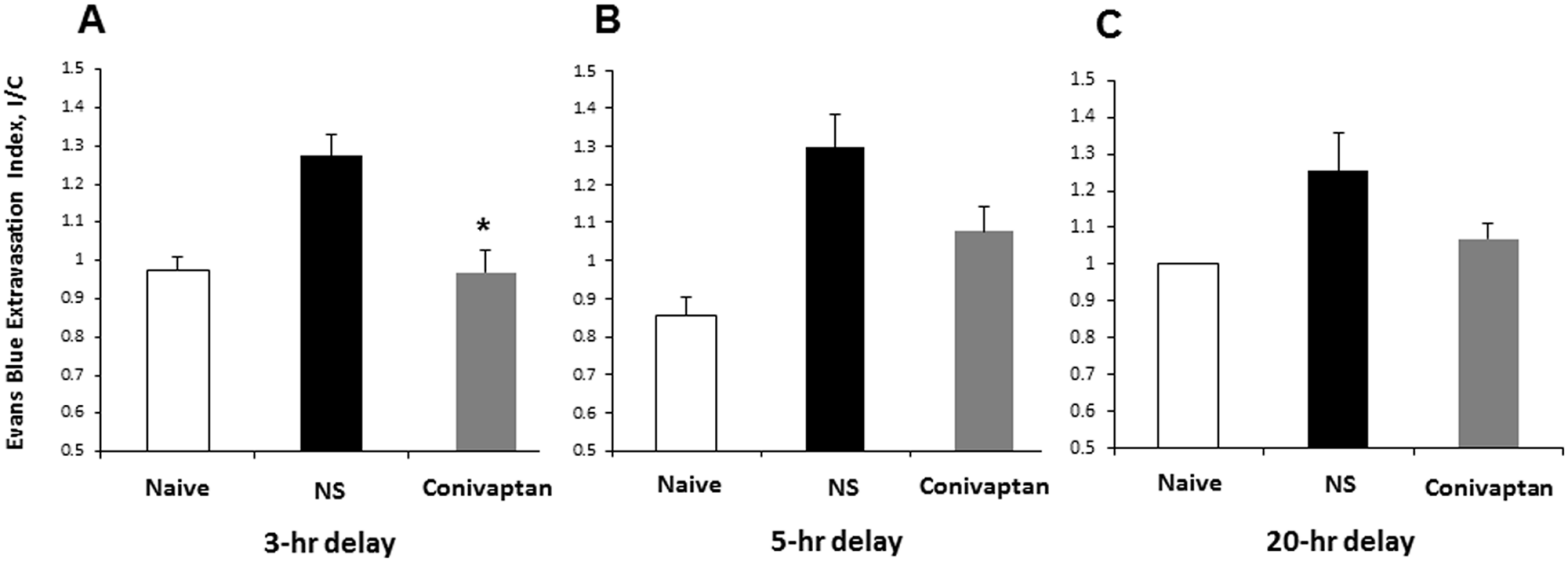
Conivaptan treatment reduces blood-brain barrier (BBB) breakdown after 3 hour delay. Evans blue extravasation was measured 48 hours after MCAO in mice treated with conivaptan or normal saline (NS) beginning at 3, 5, or 20 hours after brain ischemia. Conivaptan treatment initiated at 3 hours after MCAO (A) was effective in protecting the BBB integrity. However, 5 and 20-hour delays of treatment initiation did not produce any beneficial effect (B and C). Values are mean and SEM, *p ˂ 0.05 vs NS-treated mice, n = 5–8 per group.

Similarly, BBB integrity, assessed by EB extravasation, was protected with 3-hour delayed treatment with conivaptan, Fig 3A (p < 0.05), but further delays in treatment by 5 and 20 hours were not beneficial, Fig 3B and 3C). In the same group, there was a significant improvement in NDS when conivaptan was administered with a 3 hour delay, Table 1, (p < .05).

**Table 1:** Physiological variables.

	Vehicle 3-h delay	Conivaptan 3-h delay	Vehicle 5-h delay	Conivaptan 5-h delay	Vehicle 20-h delay	Conivaptan 20-h delay
**N**	18	18	18	18	18	18
**Body Temperature, °C:**						
**Occlusion**	36.6 ± 0.05	36.6 ± 0.04	36.7 ± 0.06	36.7 ± 0.05	36.7 ± 0.03	36.7 ± 0.04
**Reperfusion**	36.7 ± 0.04	36.7 ± 0.03	36.7 ± 0.05	36.6 ± 0.05	36.7 ± 0.04	36.8 ± 0.06
**Body Weight Loss, %**	12.5 ± 1.04	*18.9 ± 1.65	16.4 ± 1.12	15.8 ± 2.38	12.3 ± 1.98	17.1 ± 1.73
**Mortality**	3/21	2/20	3/21	3/21	4/22	3/21
**Plasma Osmolality, (mOsm/kg)**	294.2 ± 4.19	*333.4 ± 8.41	297.4 ± 3.56	*331.8 ± 7.70	295.9 ± 5.28	*339.7 ± 10.30
**Neurological Deficit Scoring, (NDS)**						
**0 hours**	2.0 (2.0–2.0)	2.0 (2.0–3.0)	3.0 (2.0–3.0)	2.5 (2.0–3.0)	2.0 (2.0–2.0)	2.0 (2.0–2.0)
**48 hours**	2.0 (1.0–3.0)	*1.0 (1.0–1.5)	2.0 (1.0–2.0)	2.0 (2.0–3.0)	2.5 (2.0–3.0)	*2.0 (1.0–2.0)

All values are mean ± SEM.

*p < 0.05 vs corresponding control.

Physiological indices, including body temperature, body weight changes and mortality rates did not differ between groups. All conivaptan-treated mice demonstrated increased plasma osmolality, Table 1.

## Discussion

Existing treatment approaches are not always effective against resistant brain edema [9] because the underlying causes are hard to identify. Many factors could impact failed attempts to reduce brain edema. For example, in addition to the pathophysiological factors and comorbidities, timing of therapy initiation can be a very influential aspect on the outcome of stroke-evoked brain edema. One significant contributor to brain edema development is often underestimated or overlooked—the syndrome of inappropriate anti-diuretic hormone release (SIADH) [7]. The effects of increased ADH (AVP) secretion on the post-ischemic brain are not fully understood, but research suggests its deleterious effects on the brain [4, 11]. Over-secretion of AVP [7] is managed by conivaptan, a drug of choice against hyponatremia and accumulation of water in the body. Owing to the V2-receptor blocking effect of conivaptan, water retention in the body [8, 12] can be reversed by inducing aquaresis and increasing plasma osmolality [13]. As a V1a receptor blocker, conivaptan prevents vasoconstriction [13] and platelet aggregation [6]. These additional qualities of conivaptan may designate it as a potential agent against stroke-evoked brain edema and BBB disruption. It is possible that aquaresis and prevention of stroke-induced vasoconstriction can broaden the use of conivaptan from being just an SIADH-treating drug.

Stroke causes early cytotoxic brain edema [14] overlapping with the BBB breakdown which instigates vasogenic brain edema [2] and hemorrhagic transformation [3]. Based on our results, conivaptan has a therapeutic window of 3 hours to reduce brain edema formation, but extended delays in therapy initiation are ineffective. Prolonged delays in conivaptan treatment may miss the opportunity to reverse ischemia-induced mechanisms already in place, which ultimately result in edema. Our study is limited and cannot confirm whether conivaptan affects specifically cytotoxic or vasogenic edema. However, ionic channels which contribute to cytotoxic edema are affected by both AVP and hypoxia [15], which may explain why AVP antagonism needs to occur within a few hours to be effective.

In this study we utilized the widely accepted MCAO model which results in brain tissue infarct reaching nearly 50% of the unilateral hemisphere by 48 hours, as previously demonstrated [16–18]. The focus of our study is limited to evaluating brain edema and BBB breakdown caused by MCAO and the ability of conivaptan to reduce those pathophysiological factors if administered at different time points. Although, the action of AVP receptors is directly linked to vasoconstriction (V1a) and water reabsorption (V2), the possibility of affecting apoptosis and necrosis may exist. Future study may be required to evaluate effects of AVP and its receptor blocker conivaptan on stroke-triggered infarct volume in the brain.

This study used a relatively short time frame and more investigation will establish if conivaptan is feasible and safe long-term. However, during the 48-hour time course, conivaptan showed that its potential vascular and aquaretic properties make it a useful candidate for therapeutic applications against stroke-induced edema and BBB disruption, extending its use from just being a drug for treatment of SIADH. The 3 hour therapeutic time window after ischemic stroke in mice presents a potential opportunity to study effects of conivaptan on post-ischemic brain edema in humans. As demonstrated by a single case report [9], the time window of conivaptan’s potential benefit against brain edema could be even longer in humans. This unlocks a new prospect for studying conivaptan in combination with tPA because the beneficial effects on BBB disruption may potentially minimize tPA-caused complications and could extend the therapeutic window for tPA administration after stroke.

## Conclusions

Conivaptan is a potent V1a and V2 receptor blocker which has the ability to reduce stroke-evoked brain edema and BBB disruption at delayed therapy initiation time points after the onset of brain ischemia in mice. However, timing of conivaptan administration is crucial for achievement of positive effects on brain edema and BBB reduction after stroke.

## Supporting information

S1 Table Tables 1–6. Raw data obtained from mice subjected to 60 minutes MCAO with reperfusion and treated with conivaptan or normal saline with a 3-, 5-, and 20-hour delay after occlusion, A, B, and C correspondingly. Brain water content (%, BWI) 1A-C; Evans Blue extravasation index ipsilateral/contralateral (I/C) 2A-C; Neurological Deficit Scoring (NDS) 3A-C; Blood osmolality (mOsmol/kg) 4A-C; Body weights (g) 5A-C; Body temperature (C°) 6A-C.
(PDF)

S1 File ARRIVE guidelines checklist. (PDF)
