# Supplementary material for: Therapeutic time window for conivaptan treatment against stroke-evoked brain edema and blood-brain barrier disruption in mice
Source: PLoS One. 2017 Aug 30;12(8):e0183985. doi: 10.1371/journal.pone.0183985 (PMC5576654; doi:10.1371/journal.pone.0183985)
Supplement: S1 Table — Raw data obtained from mice subjected to 60 minutes MCAO with reperfusion and treated with conivaptan or normal saline with a 3-, 5-, and 20-hour delay after occlusion, A, B, and C correspondingly. Brain water content (%, BWI) 1A-C; Evans Blue extravasation index ipsilateral/contralateral (I/C) 2A-C; Neurological Deficit Scoring (NDS) 3A-C; Blood osmolality (mOsmol/kg) 4A-C; Body weights (g) 5A-C; Body temperature (C°) 6A-C. (PDF) [file pone.0183985.s001.pdf]

Supporting Information: Raw Data

Tables 1A-C. Brain water content, %

A. 3-Hour Delay

Animal Groups/Numbers	NS-Ipsilateral	NS-Contralateral	Conivaptan-Ipsilateral	Conivaptan-Contralateral
1	80.77601	78.21406	79.32032	77.13787
2	79.55056	78.56709	78.0599	77.94906
3	80.36665	77.9563	79.80267	78.08843
4	81.57895	78.13316	79.52041	77.52591
5	80.73394	77.8133	80.73497	76.36868
6	79.92662	77.55627	78.54024	77.95894
7	80.06472	77.64203	79.10357	77.80013
8	79.97859	78.71131	79.18689	76.7132
9	79.51604	78.71131	79.33689	78.57143
10	79.83084	77.88204	79.08938	78.17797

B. 5-Hour Delay

Animal Groups/Numbers	NS-Ipsilateral	NS-Contralateral	Conivaptan-Ipsilateral	Conivaptan-Contralateral
1	79.83281	77.18479	78.92749	77.90769
2	79.51252	77.43468	80.80388	77.78598
3	78.20032	77.47616	78.62157	77.06699
4	81.06107	77.94994	78.46975	77.14487
5	79.07261	77.19298	77.42156	77.7027
6	78.65239	77.63006	79.65394	78.14727
7	79.05446	78.56164	77.7704	77.75956
8	78.28418	77.86537	77.36626	76.32712
9	78.2392	77.77778	78.59691	76.97161
10	80.5843	78.03571	82.2	77.60801

C. 20-Hour Delay

Animal Groups/Numbers	NS-Ipsilateral	NS-Contralateral	Conivaptan-Ipsilateral	Conivaptan-Contralateral
1	80.19462	78.49673	80.11236	78.20112
2	78.43511	78.45659	78.4324	78.26087
3	81.74727	77.82555	77.76536	76.96405
4	77.47368	77.24781	80.40897	76.74897

5	77.26081	76.84697	77.67857	77.05346
6	82.80922	77.13598	76.63344	76.29827
7	78.15073	77.07492	82.14627	77.18416
8	79.43872	77.32765	77.82178	75.84951
9	80.12821	79.25569	80.42959	77.69663
10	78.09894	77.79456	78.76254	78.53026

Tables 2A-C. Evans Blue Extravasation Index (I/C)

A. 3-Hour Delay

Animal Groups/Numbers	Naive	NS	Conivaptan
1	1	1.33903	1.195227
2	0.964366	1.080581	1
3	1.046376	1.069213	1.186721
4	0.848241	1.302084	0.915643
5	1.022662	1.237204	0.968674
6		1.326267	0.922293
7		1.270038	0.670238
8		1.566778	0.903064

B. 5-Hour Delay

Animal Groups/Numbers	Naive	NS	Conivaptan
1	0.728984	1.28444	0.917573
2	0.785483	1.422381	1.291176
3	0.815599	1.159857	1.192102
4	0.935112	1.030654	0.962656
5	1	1.850027	0.751969
6		1.272543	0.959714
7		1.206698	1.150785
8		1.032239	1.29435

C. 20-Hour Delay

Animal Groups/Numbers	Naive	NS	Conivaptan
1	1.02	1	1.013495

2	0.98	1.047619	0.956522
3	1	1.4	1.083333
4	1.01	1.090909	1.130435
5	1	1.863636	1
6		1.173913	0.913043
7		1.066667	1.208333
8		1.391304	1.24

Table 3A-C. Neurological Deficits Scoring (NDS)

A. 3-Hour Delay

Animal Groups/Numbers	NS-0h	Conivaptan-0h	NS-48h	Conivaptan-48h
1	2	3	2	1
2	2	2	1	1
3	2	3	2	1
4	2	2	2	1
5	2	2	2	1
6	2	3	0	2
7	2	3	2	2
8	2	2	2	1
9	3	2	2	1
10	2	2	1	1

B. 5-Hour Delay

Animal Groups/Numbers	NS-0h	Conivaptan-0h	NS-48h	Conivaptan-48h
1	3	3	2	2
2	3	3	1	3
3	3	3	1	2
4	3	3	1	3
5	2	3	3	2
6	3	2	2	2
7	2	2	2	2
8	2	2	2	2
9	3	2	2	3
10	2	2	2	3

C. 20-Hour Delay

Animal Groups/Numbers	NS-0h	Conivaptan-0h	NS-48h	Conivaptan-48h
1	2	2	3	1
2	2	2	3	1
3	2	2	2	1
4	2	2	0	1
5	2	2	3	2
6	2	2	3	3
7	2	2	2	2
8	2	2	4	2
9	2	3	2	2
10	2	2	2	2

Tables 4A-C. Blood Osmolality, mOsm/kg

A. 3-Hour Delay

Animal Groups/Numbers	NS	Conivaptan
1	275	263
2	311	333
3	301	305
4	272	358
5	346	311
6	297	298
7	304	304
8	249	291
9	283	287
10	302	301

B. 5-Hour Delay

Animal Groups/Numbers	NS	Conivaptan
1	275	333
2	299	347
3	289	339
4	290	309
5	303	355

6	298	325
7	297	377
8	299	331
9	317	303
10	307	299

C. 20-Hour Delay

Animal Groups/Numbers	NS	Conivaptan
1	272	338
2	301	349
3	279	354
4	276	304
5	314	376
6	291	339
7	297	398
8	301	340
9	324	303
10	304	296

Tables 5A-C. Body Weights, g

A. 3-Hour Delay

Animal Groups/Numbers	NS-0h	Conivaptan-0h	NS-48h	Conivaptan-48h
1	25	26	22	20
2	27	24	23	20
3	26	27	22	20
4	24	27	21	21
5	26	28	22	21
6	23	26	21	23
7	27	27	23	24
8	26	25	24	21
9	25	26	23	21
10	25	27	21	22

B. 5-Hour Delay

Animal Groups/Numbers	NS-0h	Conivaptan-0h	NS-48h	Conivaptan-48h

1	27	28.1	22	22.4
2	26	27.8	21	22
3	28	23.5	22	24
4	28	27.7	24	22.9
5	26	26.7	23	20.5
6	28	26	24	23
7	27	27.8	23	24.2
8	26	28.5	23	22.2
9	28.7	27.8	23	24.2
10	27	25.9	22	21

C. 20-Hour Delay

Animal Groups/Numbers	NS-0h	Conivaptan-0h	NS-48h	Conivaptan-48h
1	24	22	25	21
2	26	23	29	23
3	25	23	29	24
4	29	26	29	25
5	29	25	29	27
6	27	21	29	24
7	23	22	27	24
8	25	22	25	19
9	23	21	25	19
10	25	19	25	20

Tables 6A-C. Body Temperature, C^{o}

A. 3-Hour Delay

Animal Groups/Numbers	NS-Occlusion	Conivaptan-Occlusion	NS-Reperfusion	Conivaptan-Reperfusion
1	36.5	36.8	36.7	36.6
2	36.6	36.6	36.5	36.5
3	36.4	36.7	36.7	36.7
4	36.4	36.5	36.9	36.6
5	36.6	36.7	36.6	36.7
6	36.6	36.6	36.5	36.6
7	36.8	36.8	36.6	36.6
8	36.7	36.4	36.7	36.7
9	36.9	36.7	36.6	36.8
10	36.6	36.5	36.9	36.8

B. 5-Hour Delay

Animal Groups/Numbers	NS-Occlusion	Conivaptan-Occlusion	NS-Reperfusion	Conivaptan-Reperfusion
1	36.8	36.6	36.8	36.8
2	36.7	36.7	36.6	36.8
3	36.4	36.7	36.6	36.5
4	36.9	36.8	36.8	36.9
5	36.5	36.8	36.4	36.7
6	36.9	36.5	36.9	36.6
7	36.5	36.6	36.5	36.6
8	36.8	36.4	36.6	36.5
9	36.7	36.9	36.8	36.9
10	36.6	36.8	36.8	36.9

C. 20-Hour Delay

Animal Groups/Numbers	NS-Occlusion	Conivaptan-Occlusion	NS-Reperfusion	Conivaptan-Reperfusion
1	36.8	36.8	36.9	36.8
2	36.6	36.5	36.5	36.7
3	36.8	36.5	36.7	36.5
4	36.6	36.8	36.6	36.7
5	36.8	36.7	36.8	36.9
6	36.7	36.6	36.9	36.6
7	36.7	36.7	36.8	36.5
8	36.7	36.9	36.7	36.9
9	36.9	36.8	36.7	37
10	36.8	36.8	36.6	36.9
